# Impact of Antiviral Therapy Scale‐Up Among People Who Inject Drugs in Scotland: Regional Evidence of Hepatitis C Virus Elimination

**DOI:** 10.1111/liv.70771

**Published:** 2026-06-26

**Authors:** Norah E. Palmateer, Pantelis Samartsidis, Christopher Biggam, Andrew McAuley, Scott A. McDonald, John Dillon, Stephen T. Barclay, Samantha J. Shepherd, Rory N. Gunson, Monica Desai, Duncan McCormick, Daniela De Angelis, Matthew Hickman, Sharon J. Hutchinson

**Affiliations:** ^1^ Glasgow Caledonian University Glasgow UK; ^2^ Public Health Scotland Glasgow UK; ^3^ MRC Biostatistics Unit University of Cambridge Cambridge UK; ^4^ Incognita Data Science Trento Italy; ^5^ University of Dundee Dundee UK; ^6^ Glasgow Royal Infirmary Glasgow UK; ^7^ West of Scotland Specialist Virology Centre Glasgow UK; ^8^ UK Health Security Agency London UK; ^9^ University of Bristol Bristol UK

**Keywords:** antiviral agents, cross‐sectional studies, drug users, Hepacivirus, prevalence, preventive medicine

## Abstract

**Background:**

Expansion of highly effective direct‐acting antiviral (DAA) treatment for people who inject drugs (PWID) is regarded as essential to achieve hepatitis C virus (HCV) elimination. A decade since the introduction of DAA treatment, we aimed to assess its roll‐out and associated impact in reducing HCV prevalence among PWID in Scotland, as part of a treatment‐as‐prevention (TasP) strategy.

**Methods:**

National bio‐behavioural surveys of PWID (recruited at services providing injecting equipment) between 2010 and 2023 (*N* = 16 973), involving a questionnaire and blood spot sample (tested for HCV‐antibodies and HCV‐RNA), were used to examine treatment uptake and HCV viraemia prevalence (overall and among those with antibodies) for Tayside, Greater Glasgow & Clyde (GGC), and Rest of Scotland (RoS). We used a flexible Bayesian logistic regression model to estimate HCV prevalence and probability of having achieved an 80% reduction in HCV prevalence since 2015.

**Results:**

Between 2015–16 and 2022–23, uptake of DAAs (ever) increased 2.7‐, 5.6‐ and 4.5‐fold to 95%, 78% and 76% in Tayside, GGC and RoS, respectively. Over this period, observed viraemia prevalence declined to 4%, 16% and 15% (involving 86%, 65% and 53% decline) in Tayside, GGC and RoS, respectively. In Tayside, modelled viraemia prevalence among those with antibodies declined from 62% in 2015 to 11% in 2023, with a 53% probability that the WHO proxy target of an 80% reduction in HCV prevalence was met.

**Conclusion:**

Our study provides real‐world evidence for TasP, demonstrating that HCV elimination among PWID is feasible through community‐wide scale‐up of DAAs and population‐level monitoring surveys.

## Lay Summary

We looked at how expanding highly effective hepatitis C treatments among people who inject drugs has affected infection levels across Scotland since 2015. As treatment uptake rose sharply, the proportion of people with active hepatitis C infection fell substantially—especially in the Tayside region, where treatment scale‐up was greatest. These findings show that wide access to modern treatments can significantly reduce hepatitis C infection and support progress towards eliminating the disease in this population.

## Key Points

- HCV DAA therapy among PWID was scaled up across Scotland from 2015, with particularly high uptake in Tayside through expanded community‐based testing and treatment.
- HCV viraemia prevalence declined markedly across all regions, with the largest reduction in Tayside (86%), reaching 4% prevalence.
- Modelling indicates strong progress toward WHO elimination targets, with a 53% probability that an 80% reduction in prevalence was achieved in Tayside.
- Results provide real‐world evidence that community‐based DAA scale‐up and population monitoring can support HCV elimination among PWID.

**Abbreviations**
Ab: antibodies
CrI: credible interval
DAAs: direct‐acting antivirals
DBS: dried blood spot
GGC: Greater Glasgow & Clyde
HCV: hepatitis C virus
NESI: Needle Exchange Surveillance Initiative
NHS: National Health Service
NSP: needle and syringe provision
OAT: opioid agonist therapy
PWID: people who inject drugs
RoS: rest of Scotland
TasP: treatment‐as‐prevention
UAM: Unlinked Anonymous Monitoring
WHO: World Health Organization

## 1 Introduction

Hepatitis C virus (HCV) is a blood‐borne virus and leading cause of liver‐related morbidity and mortality worldwide, contributing to cirrhosis, liver failure and hepatocellular carcinoma. In 2022, there were an estimated 50 million people living with chronic HCV and 1 million new HCV infections globally [1]. HCV is primarily transmitted through blood‐to‐blood contact, with the majority of new infections in Europe attributable to injecting drug use via the sharing of unsterile injecting equipment [2]. In Scotland, like other high‐income countries, the largest burden of HCV infection is among people with an injecting history and people who inject drugs (PWID), with over 90% of new HCV infections occurring in this population group [3].

The World Health Organization (WHO) has set targets for elimination of HCV as a public health threat [4]. Scotland aimed to meet the WHO elimination targets by 2025, 5 years ahead of the global goal. WHO targets include an absolute reduction in incidence to ≤ 2 new annual HCV infections per 100 PWID, which is based on an 80% reduction compared to a 2015 baseline (modelled estimates indicated a global annual incidence in 2015 of 8.6/100 PWID) [5]. However, directly measuring incidence of HCV often requires large‐scale follow‐up studies, which may be challenging for many countries. While there is no official target relating to prevalence of HCV infection, this has been shown to mirror changes in transmission and may therefore provide a useful proxy for evidencing the HCV incidence target [6].

Key evidence‐based interventions for prevention of HCV transmission include sterile needle and syringe provision (NSP) and opioid agonist therapy (OAT) but these interventions are likely to yield only modest reductions in HCV prevalence among PWID over a 10‐year period [7]. In countries such as Scotland, where these interventions already have reasonably high coverage, expansion of direct‐acting antivirals (DAAs) for PWID is essential to achieve HCV elimination. Treatment‐as‐prevention (TasP)—the concept whereby treating an individual's infection prevents onward transmission—has become central to elimination strategies [8, 9].

There has been rapid scale‐up of DAAs and expansion of care pathways across Scotland, with National Health Service (NHS) Tayside (an administrative health region in Scotland) leading the way on early scale‐up in community settings including drug treatment centres, pharmacies, needle exchanges, and prisons [10]. We hypothesised that if Tayside treated at least an additional 500 PWID then chronic HCV should fall from approximately 30% to below 10% [8]. We previously reported data from 2018—midway through scale‐up in Tayside, when approximately 40% of the target (~200 PWID) had been treated in community settings [11] showing evidence of both community scale‐up of HCV treatment and rise in re‐infection rates [12].

It has now been a decade since the introduction of DAAs in Scotland. The aim of this study is to assess the implementation and impact of TasP scale‐up on HCV prevalence among PWID in Scotland, and importantly progress towards HCV elimination, using the latest data from national surveillance systems covering the period 2010–2023. Specifically, we aim to describe the trends in uptake of HCV therapy and prevalence of HCV viraemia, by Scottish region. We also aim to model HCV prevalence by region over time to determine the probability of having achieved an 80% reduction in HCV prevalence since 2015.

## 2 Materials and Methods

### 2.1 Data Sources

We used data from the Needle Exchange Surveillance Initiative (NESI), a national, biennial, bio‐behavioural survey of PWID in Scotland [13]. NESI consists of an interviewer‐administered questionnaire (to generate ‘self‐reported’ data) and dried blood spot (DBS) sample obtained from PWID attending services that provide sterile injecting equipment (covering approximately half of the 200+ services across Scotland). Data from seven NESI surveys were utilised in these analyses: 2010, 2011–12, 2013–14, 2015–16, 2017–18, 2019–20 and 2022–23. The 2015–16 data allowed us to compare progress relative to the WHO baseline, while the subsequent surveys were conducted during or after TasP scale‐up. The study was approved by the West of Scotland Research Ethics Service (reference 08/S0709/46). All participants gave their informed consent prior to their inclusion in the study.

We also included in the hierarchical model HCV prevalence and covariate data obtained from the comparable Unlinked Anonymous Monitoring (UAM) survey of PWID in England during 2010–2023. We focused on Scotland in this paper because the English results have been published previously and only the Scottish data required updating. The data for English regions are required to aid model fitting, since the smaller number of Scottish regions provides insufficient information to estimate parameters reliably. We refer the reader to Samartsidis et al. [14] for further details on the UAM survey and results of the hierarchical model for English regions.

### 2.2 Measurements

Self‐reported uptake of HCV therapy was based on participant responses to the following questions in the NESI questionnaire: ‘have you ever had drug therapy for your hepatitis C’ and ‘have you started drug therapy for your hepatitis C in the last year?’

Among those who had received therapy, the site where individuals reported commencing their therapy was established using the question ‘In relation to your most recent course of therapy, where was your therapy initiated?’ with response options as follows: hospital, prison, community (e.g., drug treatment, needle exchange), GP, or other.

Covariates used in the models included: age, sex (male/female), time since onset injecting, injected in the last 6 months (yes/no), prescribed the opioid agonist treatments methadone or buprenorphine in the last 6 months (yes/no), homeless in the last 6 months (yes/no), ever in prison (yes/no), and geographical region. Region was categorised according to the administrative health authority area (National Health Service/NHS) where a NESI respondent was recruited: Tayside, Greater Glasgow & Clyde (GGC), or Rest of Scotland (RoS). The focus on Tayside and GGC is because of Tayside's earlier and more rapid scale‐up of HCV treatment than other NHS Boards, and GGC's status as the most populous NHS board in Scotland (accounting for nearly 40% of all NESI respondents in the included survey years).

Markers of HCV infection were determined by laboratory testing of DBS for the presence of HCV antibodies (Ab) and HCV RNA. For the 2010 to 2013–14 surveys, DBS were extracted and tested for Ab in a modification of the Ortho Save 3.0 EIA [15]. From 2015 until 2020, eluted DBS were tested on the Abbott Architect i2000sr using the Architect Anti‐HCV assay; the 2022–23 samples were tested on the Abbott Alinity i. For surveys up to 2018, HCV RNA was tested using an ‘in‐house’ PCR assay using the bioMerieux extraction protocol for DBS on the Easymag and a real‐time PCR [16]. For the 2019–2020 survey, HCV RNA was extracted and amplified using a laboratory‐defined protocol on the Abbott m2000sp and m2000rt platform [17]. For the 2022–2023 survey, HCV RNA was extracted and amplified using the Abbott Alinity m platform [18].

### 2.3 Descriptive Analysis

The descriptive analysis relates to NESI data collected during 2015–2023. We calculated the proportion of respondents that self‐reported being treated for their HCV infection ever and in the last year (prior to participation in NESI). To generate appropriate denominators for these two measures of HCV therapy uptake, we created two definitions of individuals who were ‘eligible for therapy’. The denominator for those who had *ever* received therapy consisted of those with viraemia (i.e., Ab‐positive and RNA‐positive) plus those who had cleared infection with evidence of therapy (i.e., Ab‐positive and RNA‐negative and reported past therapy for HCV infection). The denominator for therapy uptake *in the last year* consisted of respondents with viraemia plus those who had cleared infection with evidence of therapy in the last year (i.e., Ab‐positive and RNA‐negative and reported therapy for HCV infection within the last 12 months).

Among those who had received therapy, we also summarised the site where individuals reported commencing their most recent episode of therapy. Sites were categorised as hospital, prison, community (including needle exchanges, pharmacies and drug treatment agencies), and general practitioner. These data were not available for the 2015–16 survey as this question was not asked.

We calculated prevalence of HCV viraemia from DBS test results as (i) the proportion that tested RNA‐positive among all respondents, and (ii) the proportion that tested RNA‐positive among the HCV Ab‐positive respondents. The latter was examined as it can reduce the risk of sampling variation over time and geographical variation by focusing on a more homogeneous population exposed to HCV infection [11, 14]. The small number of individuals with evidence of very recently acquired infection (i.e., RNA‐positive and Ab‐negative) were excluded from analyses.

### 2.4 Statistical Modelling

The statistical modelling utilises NESI data collected during 2010–2023. We updated the model described in Samartsidis et al. [14] We used Bayesian logistic regression to model prevalence (viraemia among those Ab‐positive) over time and across locations. Our model included four components: (i) a temporal trend to capture smooth variations over time; (ii) location‐specific fixed effects to adjust for baseline differences across regions (with a specified hierarchical prior on this parameter, to facilitate borrowing of information across regions); (iii) a regression component to adjust for participant characteristics (age, sex, time since onset of injecting, recent injection, prescribed OAT, homelessness, prison); and (iv) random effects to capture non‐systematic fluctuations in prevalence. Prior to 2015, the trend component was assumed to be linear and shared across locations. Post‐2015, we allowed each location to have its own trend, which is plausible due to variations in treatment uptake. We modelled these location‐specific trends using hierarchical restricted cubic splines; these allow for flexible trajectories within each location and enable partial pooling of information across regions.

For each location, we obtained the posterior distribution of prevalence over time. We used these distributions to calculate the percentage reduction between 2015 and 2023, a measure of progress towards elimination. To ensure comparability across years, we fixed covariates at their mean values (averaged over time and locations) when calculating the percentage reduction. Further, for each region, we constructed the counterfactual (i.e., the prevalence that would have been observed in the absence of TasP) by linearly extrapolating from pre‐intervention trends. The pre‐intervention period is common to all regions and is defined as the period before 2015. Visual comparison of these counterfactuals to the estimated prevalence allows us to estimate the impact of TasP on each location and year. The implicit assumption is that TasP did not influence other intervention coverage (e.g., NSP or OAT) or the covariate distributions.

To improve the precision of our prevalence estimates for Scotland, our hierarchical Bayesian model jointly analysed data from England and Scotland, enabling the NESI data to statistically borrow strength from the UAM dataset. All analyses were conducted using the nimble package in R [19, 20].

Further details of the statistical modelling are provided in Supplement S1.

## 3 Results

### 3.1 Characteristics of the Study Population

**FIGURE 1:**
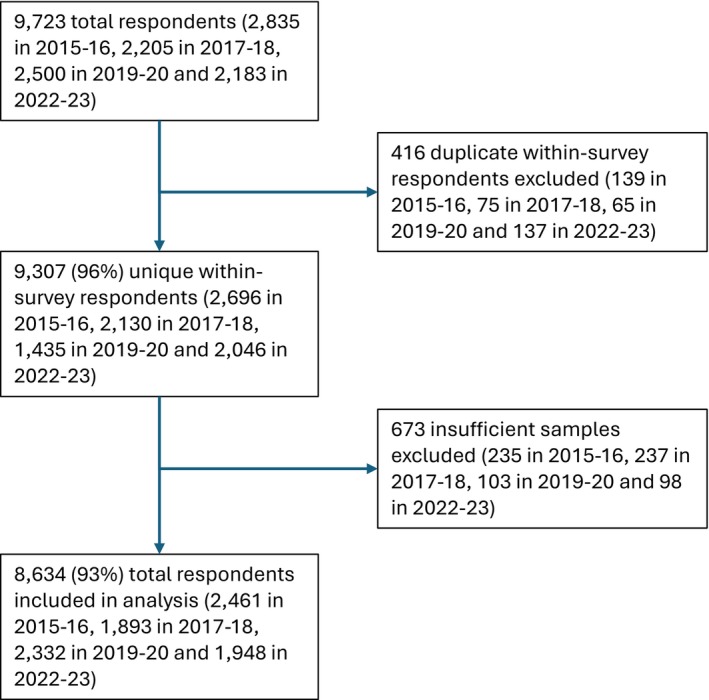
Flow chart of participants included in the descriptive analysis (2015–2023).

After removal of duplicates (respondents who had participated more than once within a given survey), data was available for 16 973 respondents surveyed between 2010 and 2023. Restricting to those surveyed between 2015 and 2023 and with a sufficient DBS sample, a total of 8634 NESI participants were included in descriptive analyses (Figure 1). Approximately 10% of participants were recruited in Tayside (range 9%–12% across survey years), 39% (range 35%–44%) from GGC, and 51% (range 44%–56%) from RoS (Table 1). Approximately 70% of respondents were male, and this was consistent across surveys. The mean age of respondents increased over time, from 38.2 in 2015–16 to 43.3 years in 2022–23. A quarter of respondents had been homeless in the last 6 months; this proportion was very comparable across the first three surveys but increased slightly (to 30%) in the 2022–23 survey. A quarter of respondents had been in prison in the last 6 months, with a small amount of variation across the surveys. The proportion who had injected powder cocaine in the last 6 months increased from 15% in 2015–16 to 63% in 2022–23. Across all surveys, more than 65% of respondents reported adequate needle/syringe coverage (i.e., at least 1 sterile needle/syringe per injection) and more than 75% had been prescribed OAT in the last 6 months. Overall, just over half (55%) of respondents tested positive for HCV antibodies.

**TABLE 1:** Sample demographics, behaviours and serological results among people who inject drugs attending injection equipment provision services in Scotland, by survey during 2015–2023 (data from the Needle Exchange Surveillance Initiative/NESI).

		2015–16		2017–18		2019–20		2022–23		Total	
		*n*	%	*n*	%	*n*	%	*n*	%	*n*	%
	Total	2461	100%	1893	100%	2332	100%	1948	100%	8634	100%
NHS Board of recruitment	Tayside	225	9%	171	9%	281	12%	186	10%	863	10%
	GGC	860	35%	756	40%	1015	44%	713	37%	3344	39%
	RoS	1376	56%	966	51%	1036	44%	1049	54%	4427	51%
Sex	Male	1736	71%	1379	73%	1673	72%	1334	68%	6122	71%
	Female	709	29%	505	27%	650	28%	583	30%	2447	28%
	Non‐response	16	1%	9	0%	9	0%	31	2%	65	1%
Age (years)	Mean (SD)	38.2 (7.2)		40.6 (7.6)		41.1 (7.8)		43.3 (8.8)		40.7 (8.0)	
Homeless in last 6 months	Yes	540	22%	442	23%	559	24%	585	30%	2126	25%
	No	1909	78%	1442	76%	1763	76%	1342	69%	6456	75%
	Non‐response	12	0%	9	0%	10	0%	21	1%	52	1%
Time since onset of injecting (years)	Mean (SD)	14.3 (8.3)		16.6 (8.8)		16.9 (9.4)		18.6 (10.5)		16.5 (9.4)	
In prison in last 6 months (i.e., released within last 6 months)	Yes	462	19%	515	27%	584	25%	446	23%	2007	23%
	No	1990	81%	1355	72%	1730	74%	1450	74%	6525	76%
	Non‐response	8	0%	23	1%	18	1%	52	3%	101	1%
Injected in the last 6 months	Yes	2009	82%	1293	68%	1575	68%	1200	62%	6077	70%
	No	450	18%	592	31%	748	32%	725	37%	2515	29%
	Non‐response	2	0%	8	0%	9	0%	23	1%	42	0%
Injected cocaine in the last 6 months^a^, ^b^	Yes	294	15%	386	30%	633	40%	759	63%	2072	34%
	No	1713	85%	902	70%	940	60%	439	37%	3994	66%
	Non‐response	2	0%	5	0%	2	0%	2	0%	11	0%
Frequency of injection in last 6 months^b^	Daily or more	1022	51%	571	44%	857	54%	565	47%	3015	50%
	Less than daily	985	49%	716	55%	713	45%	622	52%	3036	50%
	Non‐response	2	0%	6	0%	5	0%	13	1%	26	0%
Sterile NSP coverage in last 6 months^b^	100%	1439	72%	986	76%	1017	65%	809	67%	4251	70%
	< 100%	546	27%	288	22%	535	34%	326	27%	1695	28%
	Non‐response	24	1%	19	1%	23	1%	65	5%	131	2%
Prescribed OAT in last 6 months^c^	Yes	1905	77%	1590	84%	1946	83%	1549	80%	6990	81%
	No	460	19%	295	16%	379	16%	379	19%	1513	18%
	Non‐response	96	4%	8	0%	7	0%	20	1%	131	2%
Combination NSP and OAT in the last 6 months^d^	Full harm reduction	1487	60%	1343	71%	1511	65%	1232	63%	5573	65%
	100% NSP, no OAT	273	11%	172	9%	163	7%	143	7%	751	9%
	< 100% NSP, OAT	397	16%	230	12%	410	18%	259	13%	1296	15%
	< 100% NSP, no OAT	120	5%	58	3%	125	5%	67	3%	370	4%
	Did not inject, no OAT	63	3%	63	3%	88	4%	156	8%	370	4%
	Non‐response	121	5%	27	1%	35	2%	91	5%	274	3%
HCV antibody result	Positive	1343	55%	1030	54%	1264	54%	1154	59%	4791	55%
	Negative	1118	45%	863	46%	1068	46%	794	41%	3843	45%

Abbreviations: GGC, Greater Glasgow & Clyde; HCV, hepatitis c virus; NHS, National Health Service; NSP, needle and syringe provision; OAT, opioid agonist therapy; RoS, rest of Scotland; SD, standard deviation.

^a^ Includes powder cocaine and crack cocaine.

^b^ Among those who reported injecting in the last 6 months.

^c^ Methadone only for 2015–16; methadone or buprenorphine for remaining surveys.

^d^ ‘full harm reduction’ includes individuals who reported receiving OAT and either 100% NSP or did not inject.

Sample demographics and behaviours are also presented separately by region (Table S2a–c). The prevalence of HCV antibodies was higher in GGC across all survey years (range 61%–72%) as compared to Tayside (50%–52%) and RoS (47%–52%).

### 3.2 HCV Treatment Uptake and Prevalence

The proportion of respondents who had ever received therapy for their HCV infection was 95% in Tayside by 2022–23, up from 35% in 2015–16. The other regions of Scotland also saw an increase in ever uptake of therapy over time, increasing to 78% and 76% in GGC and RoS, respectively (Table 2). Receipt of HCV therapy in the last year, by contrast, increased in Tayside between 2015–16 and 2019–20 (from 23% to 53%) but then declined slightly to 42% in 2022–23. Last year therapy increased over time in GGC, from 3% to 45%, and from 8% to 37% in RoS.

**TABLE 2:** Trends in uptake of HCV therapy (self‐reported) and prevalence of HCV viraemia among people who inject drugs attending injection equipment provision services 2015–2023 (data from the Needle Exchange Surveillance Initiative/NESI).

	2015–16	2017–18	2019–20	2022–23
Therapy uptake (ever)				
All Scotland	17% (171/1006)	38% (301/785)	53% (422/802)	78% (639/815)
Tayside	35% (31/89)	65% (49/75)	76% (66/87)	95% (70/74)
GGC	14% (57/422)	31% (111/360)	58% (226/389)	78% (254/327)
RoS	17% (83/495)	40% (141/350)	40% (130/326)	76% (315/414)
Therapy uptake (last year)				
All Scotland	7% (66/949)	21% (144/680)	36% (229/643)	41% (173/426)
Tayside	23% (18/79)	43% (26/61)	53% (31/59)	42% (5/12)
GGC	3% (13/406)	16% (50/322)	42% (133/315)	45% (81/181)
RoS	8% (35/464)	23% (68/297)	24% (65/269)	37% (87/233)
Prevalence of HCV viraemia among all respondents				
All Scotland	37% (909/2461)	30% (566/1893)	19% (444/2332)	15% (286/1948)
Tayside	29% (66/225)	21% (36/171)	10% (29/281)	4% (7/186)
GGC	46% (398/860)	38% (284/756)	19% (196/1015)	16% (117/713)
RoS	32% (445/1376)	25% (246/966)	21% (219/1036)	15% (162/1049)
Prevalence of HCV viraemia among HCV Ab‐positive respondents				
All Scotland	68% (909/1343)	55% (566/1030)	35% (444/1264)	25% (286/1154)
Tayside	57% (66/115)	40% (36/89)	20% (29/145)	8% (7/93)
GGC	73% (398/542)	58% (284/491)	32% (196/620)	23% (117/513)
RoS	65% (445/686)	55% (246/450)	44% (219/499)	30% (162/548)

Abbreviations: Ab, antibody; GGC, Greater Glasgow & Clyde; HCV, hepatitis C virus; RoS, rest of Scotland.

**FIGURE 2:**
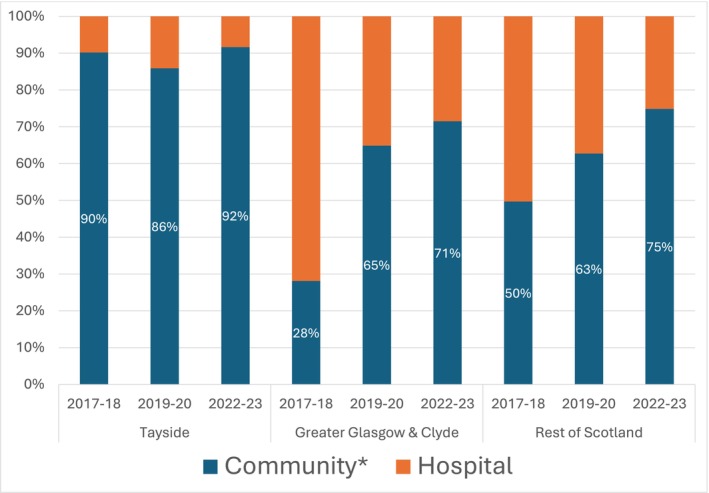
Self‐reported site of HCV therapy initiation among people who inject drugs attending injection equipment provision services, 2017 to 2023 (data from the Needle Exchange Surveillance Initiative/NESI). Data were not available for 2015–16. *Community includes the following locations: Prison, drug treatment, needle exchange, general practice and other. Drug treatment and needle exchange can include pharmacy sites. See also Table S3 for a further breakdown of the numbers and proportion of respondents reporting commencement of therapy at these sites.

The large majority (86%–92%) of respondents across all three survey years in Tayside had started treatment in a community setting (Figure 2; Table S3). In GGC, 72% of respondents had started treatment in a hospital setting in 2017–18, but this declined in 2019–20 and 2022–23, with a corresponding increase in community settings (71% in 2022–23). Similarly, 50% of respondents started treatment in community sites in RoS in 2017–18, but this increased to 75% in 2022–23.

**FIGURE 3:**
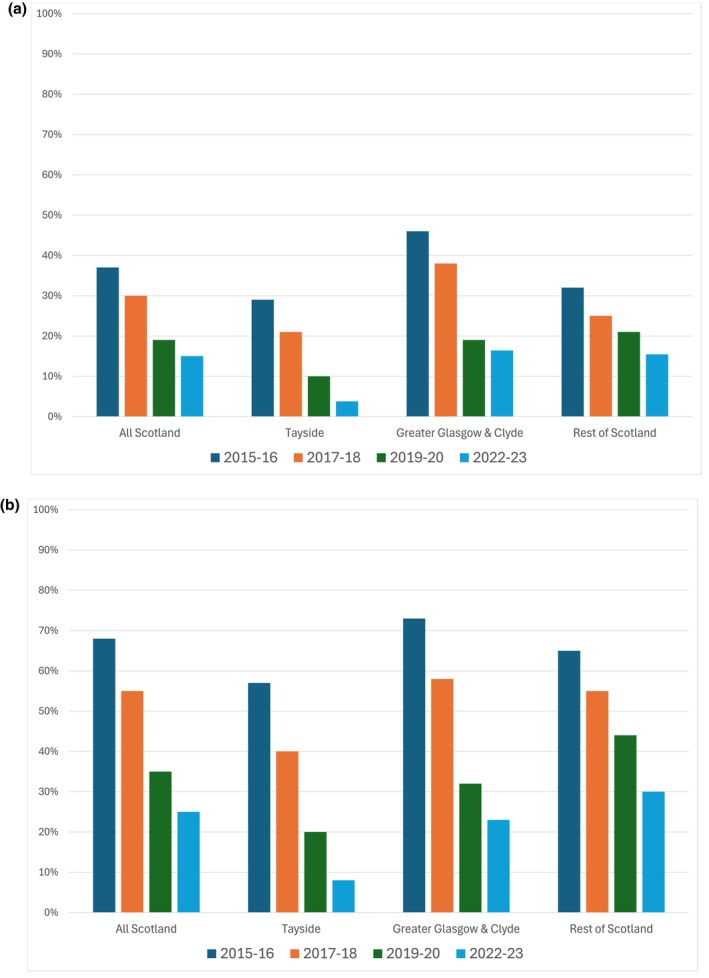
Prevalence of chronic HCV infection among people who inject drugs attending injection equipment provision services, 2015 to 2023. Figures present (a) all respondents and (b) respondents who tested HCV antibody‐positive (data from the Needle Exchange Surveillance Initiative/NESI).

The crude prevalence of HCV viraemia overall in Scotland declined from 37% in 2015–16 to 15% in 2022–23 (Table 2; Figure 3a). In Tayside, prevalence reached 4% in 2022–23, down from 29% in 2015–16 (an 86% decline). In GGC and RoS, prevalence declined by 65% (from 46% to 16%) and by 53% (from 32% to 15%), respectively. Among HCV Ab‐positive individuals, prevalence declined by 86% (from 57% to 8%), 68% (from 73% to 23%), and 54% (from 65% to 30%) in Tayside, GGC and RoS, respectively (Figure 3b).

### 3.3 Modelled Change in HCV Prevalence

**FIGURE 4:**
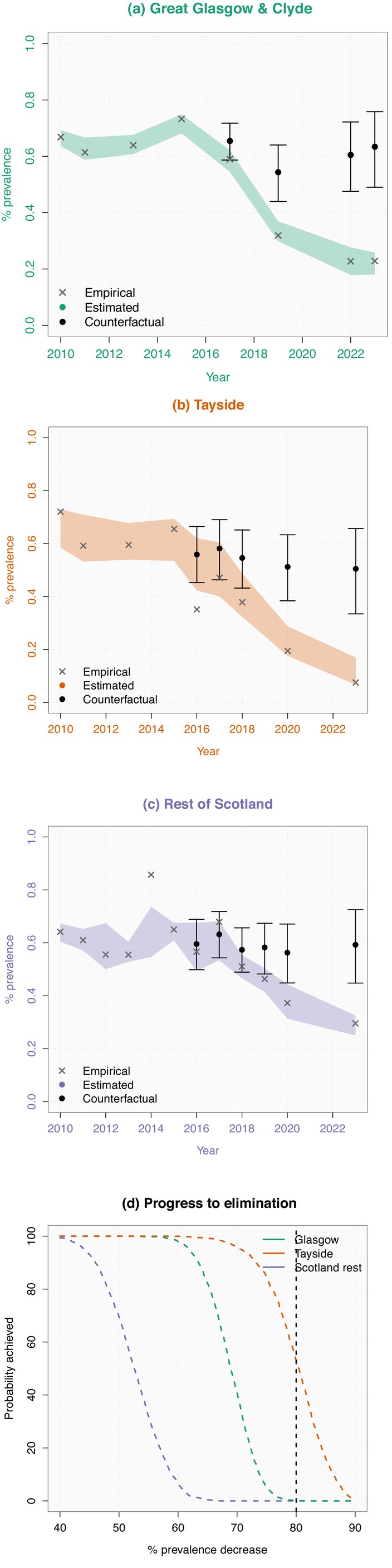
Posterior distribution of the prevalence of HCV viraemia (among HCV antibody‐positive individuals), 2010–2023, by region (panels a, b and c) and probability of achieving a relative reduction (compared to counterfactual of no intervention) in prevalence of HCV viraemia among HCV antibody‐positive individuals, by region (panel d). In panels a‐c, observed prevalence estimates in the NESI sample are represented by an ‘x’; coloured bands represent the 95% CrIs of the estimated prevalence under the intervention; and black lines indicate the 95% CrIs of the estimated prevalence in the absence of the intervention (counterfactual). In panel d, coloured lines indicate the reduction in HCV viraemic prevalence between 2015 and 2022–23. The empirical/observed prevalence is based on the calendar year of participation in the survey, and not on NESI sweeps (e.g., 2013–14, 2015–16, etc.).

Figure 4a–c shows, for the three Scottish regions, the empirical/observed prevalence (note that this is based on the calendar year of participation in the survey, and not on NESI sweeps), the posterior distributions (specifically, the 95% credible interval [CrI]) of the estimated prevalence under the intervention (i.e., scale up of TasP), indicated by coloured bands, and the counterfactuals (i.e., estimated prevalence in the absence of scale‐up), indicated by the vertical lines. The impact of TasP can be visually assessed: from approximately 2020 onwards, there is no overlap in the CrIs for the prevalence under intervention and counterfactual in all areas, indicating that there is evidence for a decrease in HCV viraemia prevalence from this date.

Point estimates for the modelled prevalence rates by Scottish region for 2015 and 2023, and the percentage reduction between 2015 and 2023, are presented in Table S4. Modelled prevalence over this period declined by 80% (from 62% to 11%), 69% (from 71% to 22%), and 52% (from 64% to 29%) in Tayside, GGC and RoS, respectively. Figure 4d and Table S4 show the probability that any given relative reduction in prevalence from 2015 to 2023 has been achieved for the three regions. The vertical dashed line (Figure 4d) indicates the WHO proxy target of an 80% reduction in HCV prevalence; there is a 53% probability that Tayside has achieved this reduction. The other regions have not achieved this reduction threshold (Table S4); however, from the plot we can see that there is a good probability (50%) that GGC and RoS have achieved a 68% and 53% relative reduction in HCV viraemia prevalence since 2015, respectively.

## 4 Discussion

We found evidence of a sustained reduction in HCV viraemic prevalence among PWID in the Tayside region of Scotland following rapid scale‐up of DAAs, commensurate with—and thus demonstrating real‐world feasibility of—reaching WHO elimination targets on treatment (90%) and reduced infection (80%) for this key population group. We also demonstrated substantial progress towards HCV elimination in the other regions of Scotland, reflecting the timing and scale of treatment expansion—these regions started from a lower baseline and had slower early scale‐up. Our findings highlight the importance of a TasP strategy to HCV elimination efforts, involving the coordinated rapid scale‐up of DAAs delivered in community‐based care pathways across multiple settings specifically designed to reach PWID [14].

To date, few countries with PWID‐focused epidemics have demonstrated HCV elimination specifically in this population group—though a range of evidence is emerging. In Iceland, early results from a national case finding, testing and treatment programme demonstrated a reduction in viraemic prevalence among PWID, from 43% to 12% between 2015 and 2017 [21]. Australia has observed significant declines in viraemia prevalence among nationwide community samples of PWID (from 24% to 17% between 2018 and 2021) coinciding with increasing treatment rates [22] and in the national survey of PWID recruited at needle exchanges (viraemia declined from 18% in 2019 to 12% in 2023) [23]. In England, Wales and Northern Ireland, the UAM survey found that HCV prevalence among PWID had declined from 26% in 2015 to 7.8% in 2023 [24]. Other, sub‐national, studies among PWID have also reported substantial reductions in chronic HCV prevalence. The DRUCK study in Germany reported a 44% decline—from 66% in 2011–2014 to 37% in 2021–2022 [25]. A study of PWID in Stockholm reported a halving of HCV prevalence, from 62% to 30% between 2013 and 2021, which they attributed to treatment scale‐up and harm reduction, although HCV incidence remained high [26]. In Norway, HCV RNA prevalence decreased from 26% in 2018 to 6.7% in 2023 among PWID recruited from low threshold services in Oslo [27, 28].

Scotland's early leadership in implementing a national hepatitis C action plan laid the foundation for coordinated prevention, testing and treatment efforts. This, alongside significant local efforts, likely enabled timely scale‐up of harm reduction and treatment‐as‐prevention strategies, supporting substantial progress towards elimination in Tayside [29]. However, our models may underestimate the true progress in Tayside, given that NESI captures high‐risk individuals and the observed prevalence is consistently on the lower side of model projections—likely because these models draw on data from areas with slower treatment scale‐up. Globally, HCV viraemia prevalence below 5% among PWID is rare and underscores the transformational impact of DAAs. No other intervention studied over the past two decades has demonstrated such rapid reductions in HCV viraemia. Our findings provide validation of empirical models that have predicted this outcome [8].

It is also important to interpret the findings in light of the COVID‐19 pandemic, which caused disruption to health and harm reduction services, including reductions in HCV testing and treatment initiation, between 2020 and 2021. As reported in other settings [30], these disruptions to services from COVID‐19 may have slowed longer‐term declines in HCV incidence. However, despite these challenges, the downward trend in HCV viraemia prevalence has continued.

Our findings also reinforce the utility of HCV viraemia prevalence as a proxy for incidence; this is particularly relevant for demonstrating progress against HCV elimination for countries that are unable to conduct adequately powered follow‐up studies to measure HCV incidence. In a recent global review and meta‐analysis of HCV incidence among PWID, only 18% of studies identified had direct measures of incidence [5]. Further, monitoring of HCV viraemia has been recommended by the EU in updated guidance, and notably, NESI is regarded as a best practice survey for monitoring drug‐related infectious diseases [31]. Serial cross‐sectional studies such as NESI not only provide evidence of changes in prevalence of chronic HCV over time but are also essential to inform parameters for modelling changes in HCV incidence [32]. However, given that these surveys (e.g., NESI, UAM) often sample higher risk PWID populations, additional evidence may be required to generate representative estimates of incidence [33].

Our analysis highlighted some demographic and behavioural shifts among PWID, including among some that were included as covariates in our models. The increasing age of NESI respondents, and corresponding increase in time since onset of injecting, reflect an ageing cohort of PWID [34]. The proportion of respondents reporting recent injecting (injecting in the last 6 months) has declined, likely because longer term injectors are more likely to be stable on OAT. Notably there has been a marked rise in the prevalence of powder cocaine injecting, a variable not included in our models. The trend is concerning because injecting cocaine is associated with more frequent injecting and therefore involves a greater risk of HCV transmission [35]. We have nevertheless demonstrated impressive reductions in HCV prevalence in this context, suggesting that TasP continues to be an effective approach to achieve elimination. However, elimination efforts will need to be cognisant of evolving drug use trends to understand their potential to affect HCV transmission and be able to adapt correspondingly. For example, Iceland has observed a high HCV reinfection rate in the context of widespread stimulant use [36].

Several limitations of this study should be noted. First, some of the NESI data (treatment uptake and covariates used in the modelling) are self‐reported and may be subject to recall or social desirability biases. However, NESI data provide a more complete picture of the cascade of care than using laboratory and treatment registers—because these data are based on a sample of PWID recruited in the community, including people who are unaware of their HCV infection. Second, this study is observational and therefore the scale or timing of exposure to the intervention could not be randomised. However, the observed reductions in prevalence are unlikely to be due to changes in other interventions—OAT and NSP—which remained at high coverage over the study period [37, 38].

In conclusion, our findings provide evidence for TasP in reducing the population prevalence of HCV viraemia to low levels among PWID commensurate with HCV elimination. The community‐based treatment approach, in concert with sustained harm reduction interventions, adopted in the Tayside region of Scotland—which has yielded an 80% reduction in the prevalence of HCV viraemia—provides a model for regions and countries with PWID‐focused epidemics to follow.

## Author Contributions

**Norah E. Palmateer:** conceptualisation, project administration, data curation, formal analysis, writing – original draft, writing – review and editing. **Pantelis Samartsidis:** conceptualisation, methodology, formal analysis, writing – review and editing. **Christopher Biggam:** project administration, data curation, writing – review and editing. **Andrew McAuley:** project administration, data curation, writing – review and editing. **Scott A. McDonald:** methodology, writing – review and editing. **John Dillon:** conceptualisation, funding acquisition, writing – review and editing. **Stephen T. Barclay:** investigation, writing – review and editing. **Samantha J. Shepherd:** data curation, validation, writing – review and editing. **Rory N. Gunson:** data curation, validation, writing – review and editing. **Monica Desai:** data curation, investigation, writing – review and editing. **Duncan McCormick:** funding acquisition, writing – review and editing. **Daniela De Angelis:** conceptualisation, methodology, writing – review and editing. **Matthew Hickman:** conceptualisation, funding acquisition, methodology, writing – review and editing. **Sharon J. Hutchinson:** conceptualisation, data curation, funding acquisition, methodology, writing – review and editing.

## Funding

This study was funded by Public Health Scotland (PHS) and the National Institute for Health Research (NIHR). NIHR funding was through the Programme Grants for Applied Research programme (Grant Reference number RP‐PG‐0616‐20 008). The study sponsors had no role in the study design, data collection, data analysis, interpretation of data, writing of the manuscript, or decision to submit the manuscript for publication.

## Ethics Statement

The authors confirm that the ethical policies of the journal, as noted on the journal's author guidelines page, have been adhered to. The study was approved by the West of Scotland Research Ethics Service (reference 08/S0709/46).

## Conflicts of Interest

Research grants from AbbVie, Bristol‐Myers Squibb, Gilead, Janssen, Merck Sharpe and Dohme, and Roche supported, in part, the scale‐up of HCV treatment in Tayside. S.T.B. has received honoraria for speaking engagements from Gilead Sciences.

## Supporting information


**Data S1:** Details of statistical modelling.

**Table S2:** Sample demographics, behaviours and serological results among people who inject drugs attending injection equipment provision services, 2015–2023 (data from the Needle Exchange Surveillance Initiative/NESI).

**Table S3:** Reported site of HCV therapy initiation among people who inject drugs attending injection equipment provision services, 2015–2023 (data from the Needle Exchange Surveillance Initiative/NESI).^a^

**Table S4:** Modelled prevalence of HCV viraemia (among HCV antibody‐positive individuals) in 2015 and 2023, percentage reduction in prevalence between 2015 and 2023, and probability that an 80% reduction in prevalence was achieved.

## Data Availability

The data that support the findings of this study are available on request from the corresponding author. The data are not publicly available due to privacy or ethical restrictions.
