# Supplementary material for: Impact of Antiviral Therapy Scale‐Up Among People Who Inject Drugs in Scotland: Regional Evidence of Hepatitis C Virus Elimination
Source: Liver Int. 2026 Jun 26;46(8):e70771. doi: 10.1111/liv.70771 (PMC13305692; doi:10.1111/liv.70771)
Supplement: Supplementary file 1 — Data S1: Details of statistical modelling. [file LIV-46-0-s003.docx]

**Supplement S1**

Details of statistical modelling

Let $y_{it}$ and $n_{it}$ be the number of PCR positive and AB positive individuals, respectively, tested in year $t=2010,\ldots,2024$ and region (Scottish board or English ODN) $i=1,\ldots,23$. We assume that $y_{it}\sim Binomial(n_{it},p_{it})$ and model the prevalence parameters $p_{it}$ using Bayesian logistic regression. Specifically, we let

$$\left( 1 \right) p_{it}=expit\left( \kappa_{i}+\lambda_{it}+X_{it}^{T}\beta+\alpha_{it} \right),$$

where $\kappa_{i}$ are region-specific fixed effects that capture systematic-over-time differences in prevalence among regions, $\lambda_{it}$ are region-specific smooth temporal trends, $X_{it}^{T}\beta$ are the regression components to adjust for sample demographics, and $\alpha_{it}$ are random effects accounting for non-systematic fluctuations in prevalence that cannot be captured by the other components. For each year and region, the covariates are: (i) % female; (ii) % injected during the past year; (iii) % on OST; (iv) % homeless; (v) average age of the sample; (vi) average injecting duration; and (vii) % with recent imprisonment.

Smoothness in the region-specific trends $\lambda_{it}$ is ensured by modelling these terms using restricted cubic splines. Specifically, for each region $i$ we assume that

$${(\lambda}_{i1},\ldots,\lambda_{iT})^{T}=Zb_{i},$$

where $Z$ is the spline basis matrix of dimension $15\times K$ ($K$ is the number of knots) that we obtain from R package mgcv, and $b_{i}$ is the vector of spline coefficients. We set the number of knots to 7, so that it is roughly 50% of the available time points. The first three knots are placed at time points 2010, 2012, 2015 and the rest are equally spaced between 2015 and 2024. We put most of them in the post-intervention period 2015-2024 as we expect trends prior to 2015 to be relatively flat and thus easy to accommodate.

To facilitate borrowing of information across the regions, and thus improve efficiency, we assign hierarchical priors to some of the model parameters. Specifically, we let $\kappa_{i}\sim Normal\left( \kappa_{0},\sigma_{\kappa}^{2} \right)$, $\alpha_{it}\sim Normal(0,\sigma_{\alpha}^{2})$ and $b_{i}\sim MVNormal(b\_0,{{(\tau}_{i}S)}^{-1})$, where $S$ is a penalty matrix obtained from the mgcv package. Standard, weakly informative priors are assigned to the remaining model parameters namely $\kappa_{0}\sim Normal(0,1)$, $\sigma_{\kappa}^{2}\sim Uniform(0.01,1000)$, $b\sim MVNormal(0,\left( \tau_{0}S \right)^{-1})$, $\beta_{j}\sim Uniform(-3,3)$ and $\sigma_{\alpha}^{2}\sim Uniform\left( 0.01,1000 \right)$.

Posterior distributions are obtained using R library nimble. The program returns $L$ Markov chain Monte Carlo samples from the posterior distribution of all parameters. Based on these, we can evaluate $p_{it}^{(l)}$, the $l$-th sample from the posterior distribution of $p_{it}$ using the transformation as shown in Equation (1). The $l$-th sample from the posterior of the % reduction is given then $100\times(p_{i,2024}^{\left( l \right)}-p_{i,2015}^{(l)})/p_{i,2015}^{(l)}$. Note that when evaluating the % reduction, we set the covariates equal to their average across regions and over time, to offset the percentage of the reduction due to change in demographics.

To construct the counterfactual (the prevalence that would have been observed if there was no scale-up of DAAs in 2015) for each region, we linearly extrapolate the values of $p_{i,2010}^{\left( l \right)},\ldots,p_{i,2015}^{\left( l \right)}$ for each region.
