# Supplementary material for: Impact of Antiviral Therapy Scale‐Up Among People Who Inject Drugs in Scotland: Regional Evidence of Hepatitis C Virus Elimination
Source: Liver Int. 2026 Jun 26;46(8):e70771. doi: 10.1111/liv.70771 (PMC13305692; doi:10.1111/liv.70771)
Supplement: Supplementary file 2 — Table S2: Sample demographics, behaviours and serological results among people who inject drugs attending injection equipment provision services, 2015–2023 (data from the Needle Exchange Surveillance Initiative/NESI). [file LIV-46-0-s002.docx]

**Supplement S2**

**Table S2.** Sample demographics, behaviours and serological results among people who inject drugs attending injection equipment provision services, 2015-2023 (data from the Needle Exchange Surveillance Initiative/NESI)

1. **NHS Tayside**

|  |  | **2015-16** | | **2017-18** | | | **2019-20** | | | **2022-23** | |  |
| --- | --- | --- | --- | --- | --- | --- | --- | --- | --- | --- | --- | --- |
|  |  | **n** | **%** | | **n** | **%** | | **n** | **%** | **n** | **%** | |
| Sex | Male | 163 | 72% | | 121 | 71% | | 193 | 69% | 123 | 66% | |
|  | Female | 60 | 27% | | 50 | 29% | | 85 | 30% | 62 | 33% | |
|  | Non-response | 2 | 1% | | 0 | 0% | | 3 | 1% | 1 | 1% | |
| Age (years) | Mean (SD) | 36.8 (7.2) | | 38.8 (8.2) | | | 39.4 (7.1) | | | 42.7 (8.5) | |  |
| Homeless in last 6 months | Yes | 40 | 18% | | 40 | 23% | | 47 | 17% | 47 | 25% | |
|  | No | 184 | 82% | | 130 | 76% | | 234 | 83% | 139 | 75% | |
|  | Non-response | 1 | 0% | | 1 | 1% | | 0 | 0% | 0 | 0% | |
| Time since onset of injecting (years) | Mean (SD) | 12.2 (8.3) | | 14.7 (9.0) | | | 15.2 (8.2) | | | 17.3 (9.3) | |  |
| In prison in last 6 months (i.e. released within last 6 months) | Yes | 48 | 21% | | 54 | 32% | | 85 | 30% | 57 | 31% | |
|  | No | 177 | 79% | | 116 | 68% | | 195 | 69% | 126 | 68% | |
|  | Non-response | 0 | 0% | | 1 | 1% | | 1 | 0% | 3 | 2% | |
| Injected in the last 6 months | Yes | 182 | 81% | | 116 | 68% | | 190 | 68% | 113 | 61% | |
|  | No | 43 | 19% | | 55 | 32% | | 91 | 32% | 72 | 39% | |
|  | Non-response | 0 | 0% | | 0 | 0% | | 0 | 0% | 1 | 1% | |
| Injected cocaine in the last 6 months ^b,c^ | Yes | 6 | 3% | | 11 | 9% | | 38 | 20% | 59 | 52% | |
|  | No | 176 | 97% | | 104 | 90% | | 151 | 79% | 53 | 47% | |
|  | Non-response | 0 | 0% | | 1 | 1% | | 1 | 1% | 1 | 1% | |
| Frequency of injection in last 6 months ^c^ | Daily or more | 94 | 52% | | 51 | 44% | | 97 | 51% | 48 | 42% | |
|  | Less than daily | 88 | 48% | | 63 | 54% | | 91 | 48% | 63 | 56% | |
|  | Non-response | 0 | 0% | | 2 | 2% | | 2 | 1% | 2 | 2% | |
| Sterile needle/syringe coverage in last 6 months ^c^ | 100% | 134 | 74% | | 82 | 71% | | 123 | 65% | 79 | 70% | |
|  | <100% | 46 | 25% | | 32 | 28% | | 66 | 35% | 28 | 25% | |
|  | Non-response | 2 | 1% | | 2 | 2% | | 1 | 1% | 6 | 5% | |

1. **NHS Tayside cont.**

| Prescribed OAT in last 6 months ^d^ | Yes | 179 | 80% | 131 | 77% | 224 | 80% | 144 | 77% |
| --- | --- | --- | --- | --- | --- | --- | --- | --- | --- |
|  | No | 39 | 17% | 40 | 23% | 57 | 20% | 41 | 22% |
|  | Non-response | 7 | 3% | 0 | 0% | 0 | 0% | 1 | 1% |
| Combination NSP and OAT in the last 6 months ^e^ | Full harm reduction | 141 | 63% | 110 | 64% | 182 | 65% | 117 | 63% |
|  | 100% NSP, no OAT | 28 | 12% | 19 | 11% | 18 | 6% | 11 | 6% |
|  | <100% NSP, OAT | 36 | 16% | 20 | 12% | 41 | 15% | 23 | 12% |
|  | <100% NSP, no OAT | 7 | 3% | 12 | 7% | 25 | 9% | 5 | 3% |
|  | Did not inject, no OAT | 4 | 2% | 8 | 5% | 14 | 5% | 23 | 12% |
|  | Non-response | 9 | 4% | 2 | 1% | 1 | 0% | 7 | 4% |
| HCV antibody result | Positive | 115 | 51% | 89 | 52% | 145 | 52% | 93 | 50% |
|  | Negative | 110 | 49% | 82 | 48% | 136 | 48% | 93 | 50% |

^a^ i.e. insufficient antibody or insufficient PCR on Ab +ves

^b^ includes powder cocaine and crack cocaine

^c^ among those who reported injecting in the last 6 months

^d^ methadone only for 2015-16; methadone or buprenorphine for remaining surveys

^e^ ‘full harm reduction’ includes individuals who reported receiving OAT and either 100% NSP or did not inject

1. **NHS Greater Glasgow & Clyde**

|  |  | **2015-16** | | **2017-18** | | | **2019-20** | | | **2022-23** | |  |
| --- | --- | --- | --- | --- | --- | --- | --- | --- | --- | --- | --- | --- |
|  |  | **n** | **%** | | **n** | **%** | | **n** | **%** | **n** | **%** | |
| Sex | Male | 618 | 72% | | 568 | 75% | | 751 | 74% | 500 | 70% | |
|  | Female | 236 | 27% | | 184 | 24% | | 260 | 26% | 203 | 28% | |
|  | Non-response | 6 | 1% | | 4 | 1% | | 4 | 0% | 10 | 1% | |
| Age (years) | Mean (SD) | 39.6 (7.1) | | 42.2 (7.0) | | | 42.1 (7.9) | | | 44.0 (9.0) | |  |
| Homeless in last 6 months | Yes | 179 | 21% | | 206 | 27% | | 301 | 30% | 305 | 43% | |
|  | No | 680 | 79% | | 543 | 72% | | 708 | 70% | 395 | 55% | |
|  | Non-response | 1 | 0% | | 7 | 1% | | 6 | 1% | 13 | 2% | |
| Time since onset of injecting (years) | Mean (SD) | 16.0 (9.0) | | 18.1 (9.1) | | | 18.5 (9.8) | | | 19.7 (11.5) | |  |
| In prison in last 6 months (i.e. released within last 6 months) | Yes | 165 | 19% | | 207 | 27% | | 257 | 25% | 148 | 21% | |
|  | No | 691 | 80% | | 536 | 71% | | 746 | 73% | 527 | 74% | |
|  | Non-response | 3 | 0% | | 13 | 2% | | 12 | 1% | 38 | 5% | |
| Injected in the last 6 months | Yes | 695 | 81% | | 513 | 68% | | 702 | 69% | 449 | 63% | |
|  | No | 164 | 19% | | 235 | 31% | | 305 | 30% | 247 | 35% | |
|  | Non-response | 1 | 0% | | 8 | 1% | | 8 | 1% | 17 | 2% | |
| Injected cocaine in the last 6 months ^b,c^ | Yes | 172 | 25% | | 254 | 50% | | 390 | 56% | 342 | 76% | |
|  | No | 523 | 75% | | 258 | 50% | | 312 | 44% | 107 | 24% | |
|  | Non-response | 0 | 0% | | 1 | 0% | | 0 | 0% | 0 | 0% | |
| Frequency of injection in last 6 months ^c^ | Daily or more | 376 | 54% | | 238 | 46% | | 394 | 56% | 239 | 53% | |
|  | Less than daily | 318 | 46% | | 275 | 54% | | 305 | 43% | 202 | 45% | |
|  | Non-response | 1 | 0% | | 0 | 0% | | 3 | 0% | 8 | 2% | |
| Sterile needle/syringe coverage in last 6 months ^c^ | 100% | 456 | 66% | | 386 | 75% | | 462 | 66% | 280 | 62% | |
|  | <100% | 228 | 33% | | 120 | 23% | | 228 | 32% | 136 | 30% | |
|  | Non-response | 11 | 2% | | 7 | 1% | | 12 | 2% | 33 | 7% | |

1. **NHS Greater Glasgow & Clyde cont.**

| Prescribed OAT in last 6 months ^d^ | Yes | 709 | 82% | 627 | 83% | 853 | 84% | 562 | 79% |
| --- | --- | --- | --- | --- | --- | --- | --- | --- | --- |
|  | No | 130 | 15% | 122 | 16% | 157 | 15% | 137 | 19% |
|  | Non-response | 21 | 2% | 7 | 1% | 5 | 0% | 14 | 2% |
| Combination NSP and OAT in the last 6 months ^e^ | Full harm reduction | 512 | 60% | 521 | 69% | 660 | 65% | 418 | 59% |
|  | 100% NSP, no OAT | 72 | 8% | 75 | 10% | 71 | 7% | 55 | 8% |
|  | <100% NSP, OAT | 187 | 22% | 98 | 13% | 180 | 18% | 112 | 16% |
|  | <100% NSP, no OAT | 33 | 4% | 22 | 3% | 48 | 5% | 24 | 3% |
|  | Did not inject, no OAT | 23 | 3% | 25 | 3% | 35 | 3% | 53 | 7% |
|  | Non-response | 33 | 4% | 15 | 2% | 21 | 2% | 51 | 7% |
| HCV antibody result | Positive | 542 | 63% | 491 | 65% | 620 | 61% | 513 | 72% |
|  | Negative | 318 | 37% | 265 | 35% | 395 | 39% | 200 | 28% |

^a^ i.e. insufficient antibody or insufficient PCR on Ab +ves

^b^ includes powder cocaine and crack cocaine

^c^ among those who reported injecting in the last 6 months

^d^ methadone only for 2015-16; methadone or buprenorphine for remaining surveys

^e^ ‘full harm reduction’ includes individuals who reported receiving OAT and either 100% NSP or did not inject

1. **Rest of Scotland**

|  |  | **2015-16** | | **2017-18** | | | **2019-20** | | | **2022-23** | |  |
| --- | --- | --- | --- | --- | --- | --- | --- | --- | --- | --- | --- | --- |
|  |  | **n** | **%** | | **n** | **%** | | **n** | **%** | **n** | **%** | |
| Sex | Male | 955 | 69% | | 690 | 71% | | 729 | 70% | 711 | 68% | |
|  | Female | 413 | 30% | | 271 | 28% | | 305 | 29% | 318 | 30% | |
|  | Non-response | 8 | 1% | | 5 | 1% | | 2 | 0% | 20 | 2% | |
| Age (years) | Mean (SD) | 37.6 (7.1) | | 39.7 (7.6) | | | 40.5 (7.7) | | | 42.9 (8.6) | |  |
| Homeless in last 6 months | Yes | 321 | 23% | | 196 | 20% | | 211 | 20% | 233 | 22% | |
|  | No | 1045 | 76% | | 769 | 80% | | 821 | 79% | 808 | 77% | |
|  | Non-response | 10 | 1% | | 1 | 0% | | 4 | 0% | 8 | 1% | |
| Time since onset of injecting (years) | Mean (SD) | 13.6 (7.7) | | 15.8 (8.4) | | | 15.8 (9.0) | | | 18.0 (9.9) | |  |
| In prison in last 6 months (i.e. released within last 6 months) | Yes | 249 | 18% | | 254 | 26% | | 242 | 23% | 241 | 23% | |
|  | No | 1122 | 82% | | 703 | 73% | | 789 | 76% | 797 | 76% | |
|  | Non-response | 5 | 0% | | 9 | 1% | | 5 | 0% | 11 | 1% | |
| Injected in the last 6 months | Yes | 1132 | 82% | | 664 | 69% | | 683 | 66% | 638 | 61% | |
|  | No | 243 | 18% | | 302 | 31% | | 352 | 34% | 406 | 39% | |
|  | Non-response | 1 | 0% | | 0 | 0% | | 1 | 0% | 5 | 0% | |
| Injected cocaine in the last 6 months ^b,c^ | Yes | 116 | 10% | | 121 | 18% | | 205 | 30% | 358 | 56% | |
|  | No | 1014 | 90% | | 540 | 81% | | 477 | 70% | 279 | 44% | |
|  | Non-response | 2 | 0% | | 3 | 0% | | 1 | 0% | 1 | 0% | |
| Frequency of injection in last 6 months ^c^ | Daily or more | 552 | 49% | | 282 | 42% | | 366 | 54% | 278 | 44% | |
|  | Less than daily | 579 | 51% | | 378 | 57% | | 317 | 46% | 357 | 56% | |
|  | Non-response | 1 | 0% | | 4 | 1% | | 0 | 0% | 3 | 0% | |
| Sterile needle/syringe coverage in last 6 months ^c^ | 100% | 849 | 75% | | 518 | 78% | | 432 | 63% | 450 | 71% | |
|  | <100% | 272 | 24% | | 136 | 20% | | 241 | 35% | 162 | 25% | |
|  | Non-response | 11 | 1% | | 10 | 2% | | 10 | 1% | 26 | 4% | |

1. **Rest of Scotland cont.**

| Prescribed OAT in last 6 months ^d^ | Yes | 1017 | 74% | 832 | 86% | 869 | 84% | 843 | 80% |
| --- | --- | --- | --- | --- | --- | --- | --- | --- | --- |
|  | No | 291 | 21% | 133 | 14% | 165 | 16% | 201 | 19% |
|  | Non-response | 68 | 5% | 1 | 0% | 2 | 0% | 5 | 0% |
| Combination NSP and OAT in the last 6 months ^e^ | Full harm reduction | 834 | 61% | 712 | 74% | 669 | 65% | 697 | 66% |
|  | 100% NSP, no OAT | 173 | 13% | 78 | 8% | 74 | 7% | 77 | 7% |
|  | <100% NSP, OAT | 174 | 13% | 112 | 12% | 189 | 18% | 124 | 12% |
|  | <100% NSP, no OAT | 80 | 6% | 24 | 2% | 52 | 5% | 38 | 4% |
|  | Did not inject, no OAT | 36 | 3% | 30 | 3% | 39 | 4% | 80 | 8% |
|  | Non-response | 79 | 6% | 10 | 1% | 13 | 1% | 33 | 3% |
| HCV antibody result | Positive | 686 | 50% | 450 | 47% | 499 | 48% | 548 | 52% |
|  | Negative | 690 | 50% | 516 | 53% | 537 | 52% | 501 | 48% |

^a^ i.e. insufficient antibody or insufficient PCR on Ab +ves

^b^ includes powder cocaine and crack cocaine

^c^ among those who reported injecting in the last 6 months

^d^ methadone only for 2015-16; methadone or buprenorphine for remaining surveys

^e^ ‘full harm reduction’ includes individuals who reported receiving OAT and either 100% NSP or did not inject
