# Supplementary material for: Impact of Antiviral Therapy Scale‐Up Among People Who Inject Drugs in Scotland: Regional Evidence of Hepatitis C Virus Elimination
Source: Liver Int. 2026 Jun 26;46(8):e70771. doi: 10.1111/liv.70771 (PMC13305692; doi:10.1111/liv.70771)
Supplement: Supplementary file 3 — Table S3: Reported site of HCV therapy initiation among people who inject drugs attending injection equipment provision services, 2015–2023 (data from the Needle Exchange Surveillance Initiative/NESI).a [file LIV-46-0-s004.docx]

**Supplement S3**

**Table S3.** Reported site of HCV therapy initiation among people who inject drugs attending injection equipment provision services, 2015-2023 (data from the Needle Exchange Surveillance Initiative/NESI)^a^

|  | Tayside | | | | | | Greater Glasgow & Clyde | | | | | | Rest of Scotland | | | | | |
| --- | --- | --- | --- | --- | --- | --- | --- | --- | --- | --- | --- | --- | --- | --- | --- | --- | --- | --- |
|  | 2017-18 | | 2019-20 | | 2022-23 | | 2017-18 | | 2019-20 | | 2022-23 | | 2017-18 | | 2019-20 | | 2022-23 | |
|  | n | % | n | % | n | % | n | % | n | % | n | % | n | % | n | % | n | % |
| Hospital | 6 | 10% | 12 | 14% | 6 | 8% | 87 | 72% | 87 | 34% | 73 | 28% | 84 | 49% | 57 | 37% | 85 | 25% |
| Prison | 6 | 10% | 9 | 11% | 10 | 14% | 18 | 15% | 23 | 9% | 49 | 19% | 29 | 17% | 34 | 22% | 76 | 22% |
| Community^b^ | 49 | 80% | 64 | 75% | 56 | 78% | 16 | 13% | 138 | 55% | 134 | 51% | 54 | 32% | 62 | 40% | 177 | 52% |
| No response | 0 | 0% | 0 | 0% | 0 | 0% | 0 | 0% | 5 | 2% | 7 | 3% | 3 | 2% | 1 | 1% | 4 | 1% |
| Total | 61 | 100% | 85 | 100% | 72 | 100% | 121 | 100% | 253 | 100% | 263 | 100% | 170 | 100% | 154 | 100% | 342 | 100% |

^a^ i.e. Restricted to individuals who reported that they had received HCV therapy in the past

^b^Includes those who responded ‘drug treatment’, ‘needle exchange’, ‘general practice’ or ‘other’
