# Supplementary material for: Impact of Antiviral Therapy Scale‐Up Among People Who Inject Drugs in Scotland: Regional Evidence of Hepatitis C Virus Elimination
Source: Liver Int. 2026 Jun 26;46(8):e70771. doi: 10.1111/liv.70771 (PMC13305692; doi:10.1111/liv.70771)
Supplement: Supplementary file 4 — Table S4: Modelled prevalence of HCV viraemia (among HCV antibody‐positive individuals) in 2015 and 2023, percentage reduction in prevalence between 2015 and 2023, and probability that an 80% reduction in prevalence was achieved. [file LIV-46-0-s001.docx]

**Supplement S4**

**Table S4.** Modelled prevalence of HCV viraemia (among HCV antibody-positive individuals) in 2015 and 2023, percentage reduction in prevalence between 2015 and 2023, and probability that an 80% reduction in prevalence was achieved.

|  | HCV prevalence 2015 ^a^ | HCV prevalence 2023 ^a^ | Percentage reduction between 2015 and 2023 ^b^ | Probability (that percentage reduction exceeds 80%) ^b^ |
| --- | --- | --- | --- | --- |
| Tayside | 62% | 11% | 80% | 53% |
| Greater Glasgow and Clyde | 71% | 22% | 69% | 0.15% |
| Rest of Scotland | 64% | 29% | 52% | 0% |

^a^ 2015 and 2023 prevalence represent point estimates that are obtained as the posterior mean

^b^ When obtaining the percentage reduction and probability, the values of the covariates are set to their mean across the study for comparability
